# Comparison of Two Fiber Post Removal Techniques Evaluating Dentin Removal, Efficiency, and Heat Production

**DOI:** 10.3390/dj13060234

**Published:** 2025-05-26

**Authors:** Matthew Fenigstein, Mazin Askar, Ahmad Maalhagh-Fard, Susan Paurazas

**Affiliations:** 1 Graduate Endodontics, University of Detroit Mercy School of Dentistry, Detroit, MI 48208, USA; fenigsmc@udmercy.edu (M.F.); askarma@udmercy.edu (M.A.); 2 Division of Clinical Dentistry, University of Detroit Mercy School of Dentistry, Detroit, MI 48208, USA; fardam@udmercy.edu

**Keywords:** fiber post, endodontic retreatment, endodontic post removal, micro-CT imaging, Munce bur, ultrasonic

## Abstract

**Background/Objectives**: The removal of a fiber post (FP) during endodontic retreatment can be the source of significant complications. This study evaluated two commonly used techniques in removing a fiber post from an endodontically treated tooth by investigating three metrics: volume of dentin removed, efficiency, and temperature increase. **Methods**: Thirty extracted, single-rooted teeth were decoronated at the CEJ, then underwent endodontic treatment and post-space preparation. Fiber posts were bonded within the canal space. Teeth were pair-matched and randomly assigned to undergo post removal via Munce bur (MB) or diamond-coated ultrasonic tip (US). Teeth were scanned with micro-CT prior to post placement and after post removal. **Results**: The volume of dentin removal was not statistically significant between groups (*p* > 0.05), but the Munce bur resulted in eccentric removal patterns. There was a statistically significant difference in the time required to remove the fiber post between MB and US (*p* < 0.05). Removal of a fiber post with a Munce bur took an average of 58 s. Removal of a fiber post with an ultrasonic tip took an average of 502 s. There was no statistically significant difference in maximum temperature generated during post removal between MB and US (*p* > 0.05). **Conclusions**: Removal of a FP with a Munce bur requires significantly less time when compared to using an ultrasonic tip, with reduced risk of generating excessive heat for either technique with adequate coolant. US can stay more centered in the canal during FP removal when compared to Munce burs, potentially reducing unfavorable outcomes.

## 1. Introduction

Endodontic therapy is indicated for a tooth with diseased dental pulp and/or apical periodontitis that results from caries or traumatic insult to the pulpal tissues [1]. If the trauma or caries eliminates significant tooth structure, an intracoronal post may be required to retain restorative material [2,3].

Fiber posts (FP) have a modulus of elasticity similar to dentin, offering improved success and survival compared to metal posts when utilized in compromised teeth [4]. FPs provide improved esthetics due to their color being similar to natural teeth [5,6]. FPs also provide the benefit of chemical bonding between the radicular dentin, luting agent, and the post [7]. For these reasons, FPs have become increasingly popular [8].

When post-treatment disease occurs [9,10] and there is an intracoronal post present, non-surgical retreatment is more challenging. Post removal can be the source of several complications, including excessive dentin removal [11], iatrogenic procedural errors [12], root perforation, microcrack formation [13], and excessive heat production [14,15]. Additionally, post removal can be time-consuming and skill-dependent [16,17].

Numerous techniques have been examined regarding post removal [18]. Fiber post removal kits have been evaluated; however, it has been shown that this technique was an aggressive technique with the most dentin removal compared to diamond bur and ultrasonic tips [19]. Ultrasonics are often utilized due to their ability to vibrate at high frequencies and operate with thin, specialized tips that allow for maximum visibility [20]. Vibrating an ultrasonic with a non-active cutting tip circumferentially around a metal post is performed to loosen the post and break the cement seal to retrieve the post. This is less predictable in the presence of a FP, as the resin structure dampens the vibrations and reduces the transmitted energy [20]. Therefore, a technique of post fragmentation is used. This may be associated with significant risk, as it requires carrying an active instrument deep into a limited canal space.

Diamond-coated ultrasonic tips or long-shanked small round burs may provide the benefit of increased visibility. Diamond-coated ultrasonic tips to remove FPs have been compared to other techniques. Using an ultrasonic tip is time-consuming, albeit useful to remove excess cement and debris from canal walls [18]. Similar findings were seen in a microCT study that compared ultrasonics and post removal kits, concluding that FP removal with ultrasonics was more time-consuming and removed more dentin volume [21]. Significant time activating an ultrasonic tip poses a risk of excessive heat production. A 10 °C temperature increase on the outer root surface causes sufficient damage to the periodontium to result in resorption and tooth ankylosis [22].

Carbide Munce exploratory burs (CJM Engineering, Ojai, CA, USA) also possess the ability to remove FPs. While operating in a slow-speed handpiece, the potential for excessive heat production may be reduced with intermittent cutting [23]. Slow-speed handpieces obstruct visualization more than ultrasonics and do not have a water port to reduce friction. Therefore, it may be technique sensitive in removing the post atraumatically.

Guided systems have also been incorporated to remove fiber posts and utilize cone-beam computed tomographic (CBCT) images and the cast surface scan to fabricate the guide. A long shank bur can then be used with the guide [24]. A sleeveless guide has also been described to remove fiber posts [25]. A Dynamic navigation system has been used with the Munce bur to remove a fiber post and found to result in less deviation and loss of tooth structure; however, the technique requires additional training and expense [26].

Minimal research exists comparing ultrasonic and Munce bur techniques of FP removal. The two techniques tested utilizing ultrasonics and Munce burs are the two most commonly used and cited techniques [26]. The use of ultrasonics is known to produce heat during metal post removal, but more data is needed to evaluate heat generation with fiber post removal. The aim of this study is to compare Munce burs and ultrasonic tips in removing FPs by evaluating the volume of dentin removal, efficiency/time required for post removal, and temperature change for each technique. The null hypothesis is that there is no significant difference in dentin removal, heat production, or working time when comparing US and MB techniques in the removal of fiber posts.

## 2. Materials and Methods

### 2.1. Selection

This study was granted exemption by the Institutional Review Board (#23-24-10) in accordance with DHHS Regulations for Protection of Human Subjects. Freshly extracted permanent teeth were collected from the Oral Surgery Department. Inclusion criteria were single-canalled teeth with fully formed apices that had not received prior endodontic treatment or received any intracanal restorations. Exclusion criteria were teeth with multiple canals, greater than 25 mm in length, carious lesions apical to the CEJ, excessively sclerotic canals, vertical root fractures, or resorptive defects. These criteria were verified via visual and radiographic examination after autoclave sterilization and storage in sterile saline [27].

### 2.2. Preparation of Samples

Teeth that met the inclusion criteria were decoronated at the level of the CEJ with an Endo Z bur (Kerr Corporation, Brea, CA, USA). Teeth were pair-matched based on length and width of tooth structure and randomly divided into each experimental group: A single operator performed the procedures with the aid of a chairside assistant. Blinding was not done since the standardized samples were already randomly sorted into groups.

Munce bur (MB) and ultrasonic (US) root canals were scouted with #10 K-File (Kerr Corporation, Brea, CA, USA), and the working length was determined and confirmed with digital radiograph (Schick 33, Dentsply Sirona, Charlotte, NC, USA).

The following rotary instrumentation technique was implemented: ProTaper Sx (Denstply Sirona USA, Charlotte, NC, USA) → 17.04 → 25.04 → 30.06 → 35.06 (EdgeEndo, Albuquerque, NM, USA), with 3 mL saline irrigation and recapitulation with #10 K-File. Following instrumentation, teeth were irrigated thoroughly with 3 mL each of 6% NaOCl, 17% EDTA, 6% NaOCl and dried with paper points (EdgeEndo, Albuquerque, NM, USA). Roots were obturated with corresponding gutta percha cones (EdgeEndo, Albuquerque, NM, USA) and BC Sealer (Brasseler USA, Savannah, GA, USA). A System B tip (Kerr Corporation, Brea, CA, USA) was pre-measured to 6–7 mm from working length and activated at 200 °C to remove the coronal gutta percha [28]. The post space length was 10 mm for all the canals. A post space was then created utilizing a 3M™ RelyX™ 3D Glass FP Drill (3M, St. Paul, MN, USA) that corresponded to the size of 1/3 the width of the root [29] when measured with a periodontal probe.

### 2.3. Initial Micro-CT Scan

**Figure 1:**
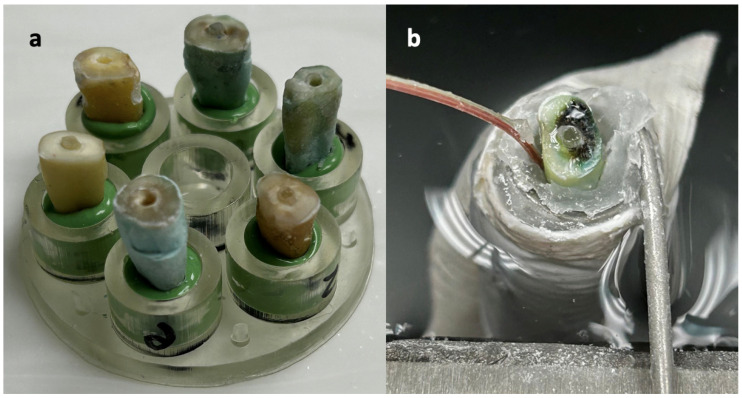
(**a**) 3D-printed jig to hold samples in the micro-CT unit. Aquasil PVS was used to maintain teeth in identical orientation during removal and replacement. (**b**) Sample prepared for post removal, secured in place in a water bath with a temperature probe affixed to the lateral root surface.

A mounting jig was printed from stereolithography (.stl) files provided by the University of Michigan MicroCT Core (mCT100, Scanco Medical, Wangen-Brüttisellen, Switzerland) to secure specimens within the micro-CT unit. Each tooth was set in an individual well with Aquasil PVS monophase (Dentsply Sirona, Charlotte, NC, USA) to allow removal and replacement in the same orientation (Figure 1a). An initial micro-CT to obtain a baseline dentin volume was conducted at the University of Michigan MicroCT Core Lab (Ann Arbor, MI, USA) with the following settings: voxel size 25 µm, 90 kVp, 155 µA, 0.1 mm CU filter, and integration time 500 ms.

### 2.4. Post Placement and Removal

The corresponding post (3M, St. Paul, MN, USA) was verified by radiograph to be in contact with the apical gutta percha. Posts were luted using 3M™ RelyX™ Unicem 2 Self-Adhesive Resin Cement (3M, St. Paul, MN, USA) according to the manufacturer’s DFU and segmented to ~2 mm above the CEJ with Endo Z bur [30] (Kerr Corporation, Brea, CA, USA). The cement was allowed to set 24 h.

Teeth were secured in a water bath (Thermo Fisher Scientific, Waltham, MA, USA) maintained at 37 °C throughout the study. A temperature probe (Omega Engineering, Stamford, CT, USA) was affixed to the sample at the mid-root level with rope wax (Hanau, Germany) and connected to a 0.04% accuracy digital thermometer (Omega Engineering, Stamford, CT, USA) (Figure 1b).

All post removal protocols were completed by a single operator, aided by a chairside assistant. Posts were removed with a BUC-1 and -3 ultrasonic tip (US) (Kerr, Brea, CA, USA) or a size 3 Munce discovery bur (MB) (KJM Engineering, Ojai, CA, USA) operated in an electric handpiece at 3500 RPMs. The ultrasonic unit (Acteon, Paris, France) was operated at 65% power. This protocol was performed under a Leica M320 dental operating microscope (Leica Microsystems, Wetzlar, Germany) at 10–16× magnification. During operation, a chairside assistant provided intermittent air/water bursts through a Stropko irrigator (Ultradent, South Jordan, UT, USA). When debris accumulated, the tip or bur was withdrawn and wiped clean with alcohol-soaked gauze. Data was recorded beginning with the initial contact of the post until the gutta percha could be visualized. Each bur/tip was used for three trials, then discarded.

### 2.5. Follow-Up Micro-CT Scan

To evaluate dentin removal, samples were replaced in their respective wells and re-scanned with the micro-CT at the same settings and orientation. A comparison was made before and after instrumentation to measure changes in canal volume in mm^3^. Micro-CT images were constructed with pre-instrumentation (green) and post-instrumentation (pink) geometry.

The two comparative scans were taken in identical orientation. The volumetric scans were then overlaid on one another, and the software performed volumetric analysis. This analysis is able to calculate the volume of dentin that was removed during post removal in comparison to the volume of the dentin prior to removal of the post.

### 2.6. Statistical Analysis

To evaluate temperature change and efficiency, *T*-Tests and Mann–Whitney U Tests were conducted, in addition to calculating traditional means and standard deviations. Micro-CT analysis was performed using the manufacturer’s evaluation software, IPL Register v5.42: (Scanco Medical, Wangen-Brüttisellen, Switzerland). The produced images were superimposed before and after post removal, enabling three-dimensional visualization and 3D quantification of the areas of dentin removal. Images of each specimen were reconstructed (Scanco manufacturer’s microCT software v4.2) for 3D volume, surface area, and SMI evaluation.

## 3. Results

### 3.1. Dentin Removal

**Figure 2:**
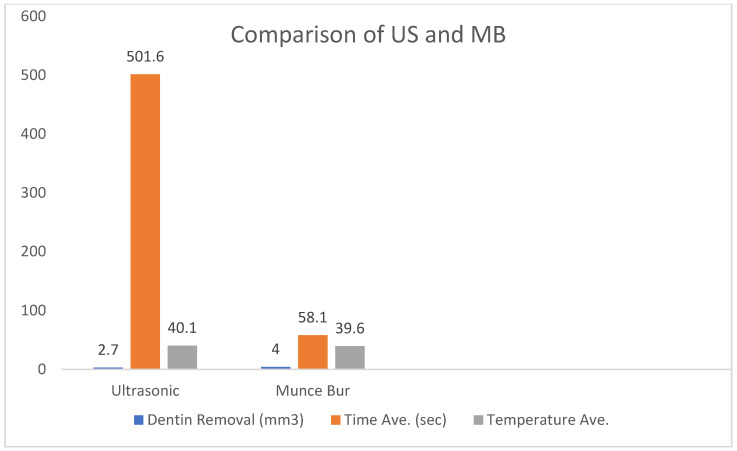
Comparison of US and MB groups regarding dentin removal, US 2.7 mm^3^, MB 4.0 mm^3^ (*p* > 0.05), Average time for removal US 501.6 s, MB 58.1 s (*p* < 0.001), Average maximum temperature US 40.1, MB 39.6 (*p* > 0.05).

**Figure 3:**
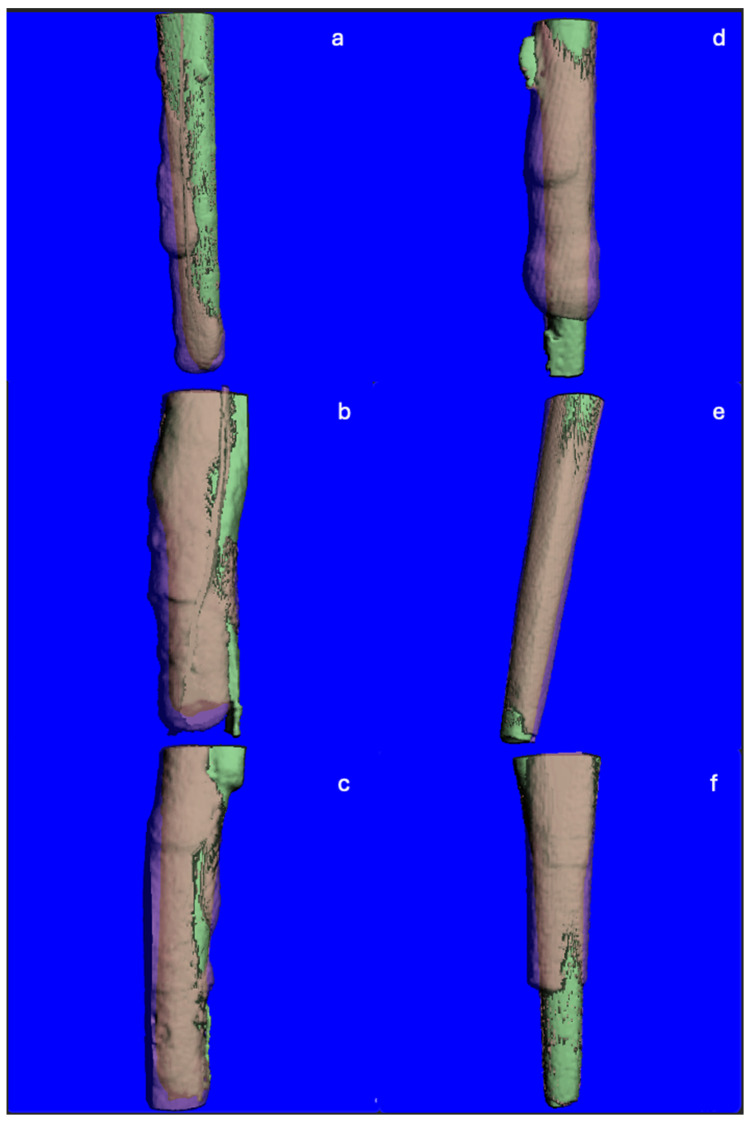
TIF images created from ScanCo Software v4.2. Superimposed micro-CT slices showing the pre- (green) and post- (pink) instrumentation root canal spaces. (**a**–**c**) Representative images from the MB group, showing dentin removal biased to one side of the canal. (**d**–**f**) Representative images from the US group, showing concentric dentin removal maintained within the center of the canal.

The mean change in canal volume for US was 12.6 mm^3^ to 15.3 mm^3^. The mean change in canal volume for MB was 11.1 mm^3^ to 15.1 mm^3^. This overall change in canal volume was found to be statistically significant (*p* < 0.001). However, when this was compared between the two techniques, there was no significant difference (*p* = 0.248) (Table 1, Figure 2). Despite this, a difference in patterns of dentin removal was realized between the modalities. US displayed a pattern of removal that was maintained within the center of the canal. MB resulted in eccentric dentin removal, deviating towards the side of the root (Figure 3).

### 3.2. Efficiency

On average, post removal required 58.1 s for MB (Std. dev. = 31.0), compared to 501.6 s (Std. dev. = 229.8) for US. This was found to be statistically significant (*p* < 0.001). The range of time required to remove the fiber post in the US group was greater than that of the MB group. The minimum and maximum time to remove a post with US was 182 and 951 s, respectively. Alternatively, the minimum and maximum time to remove a post with MB was 31 and 150 s (Table 1, Figure 2).

### 3.3. Temperature Change

Neither technique in any of the trials, with the exception of one outlier in the US group, produced an increase in temperature that surpassed 10 °C above body temperature. The maximum temperature that was reached across both groups with proper coolant was 42.9 °C, occurring in MB. The maximum temperature reached in the US was 42.2 °C. The average maximum temperature produced for the US and MB was 39.6 °C and 40.1 °C, respectively. There was no significant difference in the average maximum temperature between the two groups based on an independent *t*-test (*p* = 0.60) (Table 1, Figure 2).

## 4. Discussion

This study aimed to compare two techniques in removing a FP. Minimizing the amount of dentin removal during fiber post removal is important as excessive removal can directly lead to both immediate and long-term unfavorable outcomes, such as perforation and vertical root fracture [31]. There was no significant difference in the volume of dentin removed between US and MB. However, when viewing the superimposed TIF images for each trial, a difference in the pattern of dentin removal was observed (Figure 3). US resulted in dentin removal that was maintained within the center of the root, whereas MB resulted in eccentric dentin removal. This was likely due to the Munce bur’s ability to cut through the post and dentin. This resulted in perforation of the root in a MB trial. Using the ultrasonic tip required slow, diligent cutting that allowed distinct visualization between the post and the root dentin. Despite the volume change being similar between MB and US, using an ultrasonic to remove a FP may be safer than a Munce bur to avoid perforation and excessive dentin removal. Eccentric cutting may occur as a result of angulation of the bur, difficulty in access, visualization, and estimating the angulation of the post [26]. Operator orientation may also play a role. Alfadda et al. note that working within a limited anatomic area may affect proper visualization, and operator experience may also influence removal of dentin during post removal [24]. A study evaluating fiber post removal utilizing three techniques of post removal kit, ultrasonic tip, and long shank round bur found that deviation from the original root canal occurred in all three groups [32]

Minimizing treatment time is important for both the patient and the provider. It took considerably more time to remove a FP with an ultrasonic compared to a Munce bur. This is consistent with previous findings of a a systematic review that concluded that ultrasonics tend to have a longer working time when compared to other methods [18]. It was also noted that this may be explained by the energy from the ultrasound being absorbed by the adhesiveness of the resin systems. This could result in increased time required for removal with ultrasonics.

Greater variability of time to remove the FP was seen in the US group. Using the ultrasonic tip required slow, careful cutting to ensure distinct visualization between the post and the root dentin. Factors that may contribute to variability are intermittent air/water bursts, possibly impairing visibility and resulting in increased time. The number of times the ultrasonic tip was used as well as the time for debris removal, may also have impacted the wider standard deviation in the US group.

Differences in time to remove FP with ultrasonic may also be attributed to operator experience. In the current study, all specimens were managed by a single operator, who may have become more efficient as the procedure progressed. An endodontic resident completed all procedures. Level of experience has been cited in a study that noted significant differences in time to remove FP based on operator experience, whether being a general dentist or endodontist with 10 years of experience [16]. In another study, when comparing a specialist with a general dentist in the time required to remove a fiber post, the operator with less experience removed the post more cautiously, so as not to remove excess dentin. This could translate into increased variability for removal time [33].

Ultrasonics or Munce bur produced a significant temperature increase above 10 °C. It was demonstrated in this study that using coolant avoids deleterious temperature production. Air and water spray prevented both the US and MB from reaching this threshold during FP removal. Literature demonstrates the high heat generation potential of ultrasonic use during metal post removal [14,15,34], which is consistent with findings evaluating dry ultrasonic use during FP removal [34]. A study comparing heat generation with the removal of fiber posts with US tips with and without cooling, noted significantly higher temperatures with 40 s of dry cutting [35]. One US trial in this experiment was performed with minimal coolant, which resulted in a temperature of 51.4 °C. The water port available on many ultrasonic tips may be insufficient or can obscure vision. Therefore, a chairside assistant is recommended to provide supplementary coolant and air spray to clear the field and keep temperatures at a safe level.

Extracted teeth were utilized to evaluate temperature change and dentin removal. The authors acknowledge study limitations. Specimens could not be entirely standardized, but were pair-matched according to inclusion criteria to minimize variability.

The economics of FP removal may also play a role in deciding which technique to employ. Munce burs are inexpensive compared to the BUC ultrasonic tips. Although this was not an investigation into the durability of each of these cutting tools, it was clear that the Munce bur maintained its performance after the third trial, whereas the ultrasonic tip was visibly worn. This played a role in the inefficiencies of the ultrasonic group; however, even as new tips were introduced every three trials, the most efficient ultrasonic trial was still more time-consuming than the slowest MB trial. Future studies could explore US and MB techniques with guided techniques on the removal of fiber posts on multi-rooted teeth.

## 5. Conclusions

Within the limitations of this study, US and MB removed a similar volume of dentin during FP removal; however, the use of a Munce bur resulted in more eccentric dentin removal that may result in weakened dentin structure. Munce burs are significantly more efficient than an ultrasonic tip in removing a FP from an endodontically treated tooth. With adequate coolant technique, neither ultrasonic nor Munce bur produced a temperature that surpasses deleterious thresholds.

## Figures and Tables

**Table 1:** A summary of the results found during the fiber post removal trials *p*-values < 0.05 were considered statistically significant.

	Munce Bur	Ultrasonic	*p* Value
**Dentin Removal**			
Average pre-operative canal volume (mm^3^)	11.1	12.6	0.248
Average post-operative volume (mm^3^)	15.1	15.3	
**Efficiency**			
Average time for post removal (s)	58.1	501.6	<0.001
Minimum time for removal (s)	31	182	
Maximum time for removal (s)	150	951	
**Temperature Change**			
Group Maximum Temperature (°C)	42.9	42.7	0.60
Average Maximum Temperature (°C)	39.6	40.1	

## Data Availability

Data is unavailable due to privacy.
